# Metabolism Study of Notoginsenoside R_1_, Ginsenoside Rg_1_ and Ginsenoside Rb_1_ of Radix Panax Notoginseng in Zebrafish

**DOI:** 10.3390/molecules16086621

**Published:** 2011-08-05

**Authors:** Yingjie Wei, Ping Li, Hongwei Fan, Yunru Peng, Wei Liu, Changmei Wang, Luan Shu, Xiaobin Jia

**Affiliations:** 1 Key Laboratory of New Drug Delivery System of Chinese Materia Medica, Jiangsu Provincial Academy of Chinese Medicine, 100 Shizi Street, Nanjing 210028, China; Email: wyj970@163.com (Y.W.); pengyunru@yahoo.com.cn (Y.P.); sophia.liuwei@qq.com (W.L.); hanxi186@yahoo.com.cn (C.W.); shuluan2006@hotmail.com (L.S.); 2 Key Laboratory of Modern Chinese Medicines and Department of Pharmacognosy, China Pharmaceutical University, 24 Tongjia Lane, Nanjing 210009, China; Email: liping2004@126.com; 3 Nanjing Medical University, Affiliated Nanjing First Hospital, Lab of Clinical Pharmacology, 68 Changle Road, Nanjing 210006, China; Email: fanhongwei178@sina.com

**Keywords:** zebrafish, notoginsenoside R_1_, ginsenoside Rg_1_, ginsenoside Rb_1_, metabolism

## Abstract

Zebrafish, a common model organism for studies of vertebrate development and gene function, has been used in pharmaceutical research as a new and powerful tool in recent years. In the present study, we applied zebrafish for the first time in a metabolic study of notoginsenoside (R_1_), ginsenoside (Rg_1_) and ginsenoside (Rb_1_), which are saponins isolated from Panax notoginseng. Metabolites of these three saponin compounds in zebrafish after exposure for 24 h were identified by high performance liquid chromatography - electrospray mass spectrometry (HPLC-ESI-MS) with a Zorbax C-18 column for separation using a binary gradient elution of 0.05% formic acid acetonitrile - 0.05% formic acid water. The quasi-molecular ions of compounds were detected in negative mode. Step-wise deglycosylation metabolites and hydroxylation metabolites of the three saponins were found, which were coincide with regular methods for metabolic analysis. Our study demonstrated that the zebrafish model can successfully imitate the current metabolic model with advantages of lower cost, far less amount of compound needed, easy set up and high performance. Our data suggests that the zebrafish metabolic model has the potential for developing a novel method for quickly predicting the metabolism of Chinese herb components, including those of trace compounds.

## 1. Introduction

In recent years, metabolic studies of Traditional Chinese Medicine (TCMs) have been applied effectively in the major fields of TCM research, including material basis, mechanism of action and quality control. However, there are still limitations for metabolic study methods so far using either *in vivo* or *in vitro* models in TCM research: the *in vivo* method is resource costly, involving consumption of a great amount of compound which is difficult to purify from Chinese herbs, and many trace components cannot even be evaluated. On the other hand, the *in vitro* method is expensive due to the high standard of experimental conditions, which makes it hard to perform in some laboratories. Therefore, it is really important to establish an alternative metabolic study method for TCMs, which would combine advantages of these methods and overcome compound shortages to make it available for even trace compounds analysis.

Over the past ten years, zebrafish has been used as a popular model organism in diverse fields of research such as developmental and evolutionary biology, toxicology and pharmacology. They are considered a new powerful tool, especially in pharmaceutical research [1,2,3,4,5,6,7], because they are genetically similar to humans and have the same complex organs found in mammals. Besides, it is known there are intestinal bacteria in zebrafish [8] and P450s enzymes are expressed [9,10], which make them suitable for study of drug metabolism. Based on above advantages of zebrafish, we put forward the ideas for metabolic study of TCMs using zebrafish for the first time [11,12].

**Figure 1:**
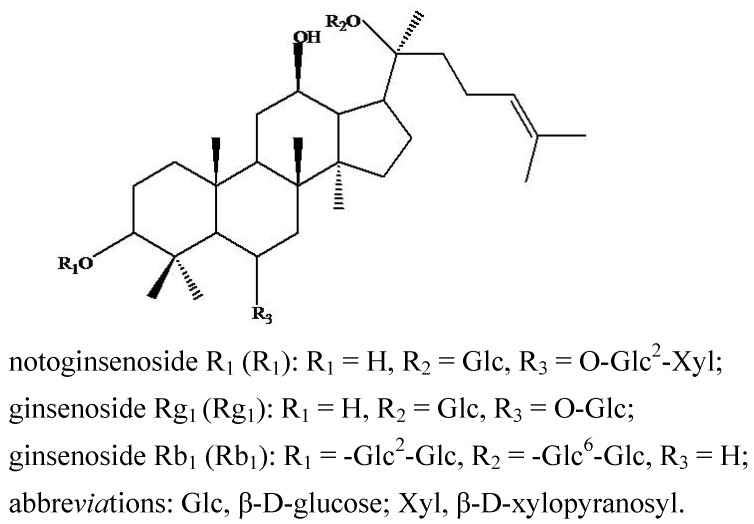
Structures of notoginsenoside R_1_, ginsenoside Rg_1_ and ginsenoside Rb_1_ in Radix Panaxnotoginseng in this study.

Panax notoginseng, a well known Chinese medicinal herb named Sanqi, has been widely used for the treatment of analgesia, hemostasis, cardiovascular and cerebrovascular diseases. Notoginsenoside R_1_ (R_1_), ginsenoside Rg_1_ (Rg_1_) and ginsenoside Rb_1_ (Rb_1_) (Figure 1) are the major bioactive saponins of Sanqi monitored for quality control [13]. A number of metabolic studies of these three saponins *in vivo* or *in vitro* have been reported together with their metabolic mechanisms, which were demonstrated to involve stepwise deglycosylation and hydroxylation. For example, metabolites of R_1_ are ginsenoside Rg_1_, notoginsenoside R_2_, ginsenoside Rh_1_ or F_1_, protopanaxatriol (ppt) and hydroxynotoginsenoside R_1_ [14,15,16]; metabolites of Rg_1_ are ginsenoside Rh_1_ or F_1_, protopanaxatriol (ppt) [17,18,19,20,21,22]; and metabolites of Rb_1_ are ginsenoside Rd, ginsenoside Rg_3_ or F_2_, ginsenoside Rh_2_, hydroxyginsenoside Rb_1_ and protopanaxadiol (ppd) [23,24,25,26,27,28]. For the first time, we have investigated a new method using zebrafish as a model in the metabolic study of TCM components due to the advantages of zebrafish. In the present study, notoginsenoside R_1_, ginsenoside Rg_1_ and ginsenoside Rb_1_ whose metabolic mechanisms have been clearly elucidated were selected as sample compounds, and high performance liquid chromatography—electrospray mass spectrometry (HPLC-ESI-MS) was used for analysis of their metabolites after zebrafish exposure.

Based on results from zebrafish metabolic study model and existing *in vivo* and *in vitro* study methods, we report evidence for a feasible and economic model, and our research should contribute to developing a novel, simple, low cost, high-performance metabolic study model for trace components of TCMs.

## 2. Results and Discussion

### 2.1. Conditions Selected for Zebrafish Metabolism Experiments

Method for drug administration and sampling: due to the small size of zebrafish, it is difficult to administer small quantities of drugs and to obtain blood samples for trace component analysis. But if zebrafish are exposed to drug solutions, they can absorb compounds from solution, and the metabolites transformed by zebrafish will be continuously output into the solution, so analysis of component changes in solution and whole zebrafish bodies can provide some information about drug metabolism, and this method is simple and feasible.

Solution concentration of compounds should not influence zebrafish activity for at least 24 h; the water temperature should be controlled within a suitable living temperature for zebrafish (from 20 °C to 26 °C; the present experiments were performed in a 23 °C thermostated waterbath); taking factors such as the stability of blank drug solution and zebrafish activity into consideration, and referring to commonly sampling within 24 h of mammalian metabolism experiments, we selected 24 h as sampling time after zebrafish exposure to compounds, thus the accumulation of metabolites may satisfy the demand for detection, and usage amount of compounds would be minimized. The R_1_, Rg_1_ and Rb_1_ required in the present test was only 9.63, 16.75 and 21.24 µg/mL, respectively.

### 2.2. Analysis of Metabolic Components of Notoginsenoside R_1_, Ginsenoside Rg_1_ and Ginsenoside Rb_1_ after Zebrafish Exposure by HPLC-ESI-MS

R_1_, Rg_1_, Rb_1_ and their metabolites after zebrafish exposure were identified by HPLC-ESI-MS with an ESI source in negative mode. Consistent with reference [29] the saponins and their metabolites exhibited their quasi-molecular ions of [M−H]^−^ and [M+HCOO]^−^ for molecule mass information. By extracting the ion current, attentive study of the mass spectra of compounds and comparison with reference data and some standards, both stepwise deglycosylation and hydroxylation metabolites of R_1_, Rg_1_ and Rb_1_ were identified by comparing with blank samples. 

**Figure 2:**
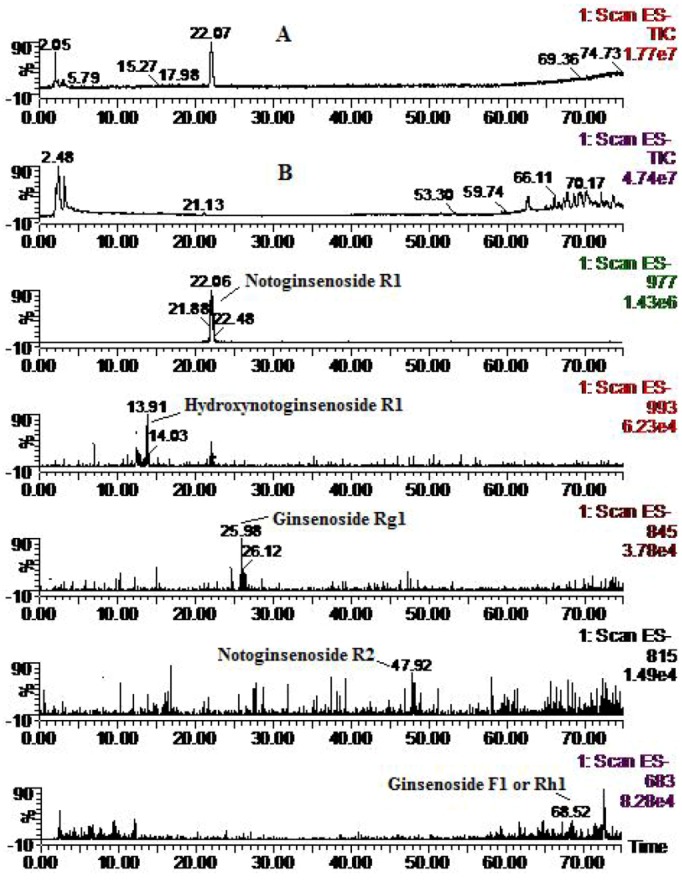
Total ion chromatogram (**A**: solution; and **B**: zebrafish) and extracted ion chromatograms for notoginsenoside R_1_ after zebrafish exposure for 24 h.

Four metabolites of R_1_ were identified, in addition to the parent component notoginsenoside R_1_ (MW 932) (Figure 2), including hydroxynotoginsenoside R_1_ (MW 948), notoginsenoside R_2_ (MW 770) degradation products derived from R_1_ *via* cleavage of one molecule of glucose, ginsenoside Rg_1_ (MW 800) derived from R_1_ *via* cleavage of one xylose moiety, and ginsenoside F_1_ or Rh_1_ (MW 638), monoglucosylated protopanaxatriol ginsenoside, derived from R_1_ *via* cleavage of both glucose and xylose moieties. These results were consistent with previous reports on mammalian metabolism [14,15,16].

**Figure 3:**
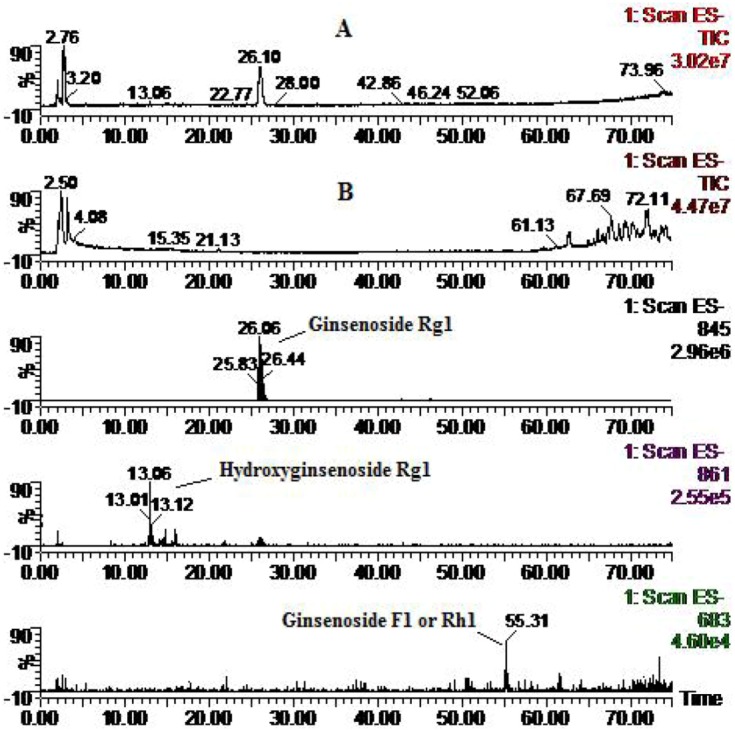
Total ion chromatogram (**A**: solution; and **B**: zebrafish) and extracted ion chromatograms for ginsenoside Rg_1_ after zebrafish exposure for 24 h.

Two metabolites of Rg_1_ were identified, in addition to the parent component ginsenoside Rg_1_ (MW 800) (Figure 3), including hydroxyginsenoside Rg_1_ (MW 816), and a monoglucosylated protopanaxatriol ginsenoside named ginsenoside F_1_ or Rh_1_ (MW 638) derived from Rg_1_ *via* cleavage of a glucose moiety. These results are consistent with previous reports concerning the rat metabolism [17,18,19,20,21,29].

**Figure 4:**
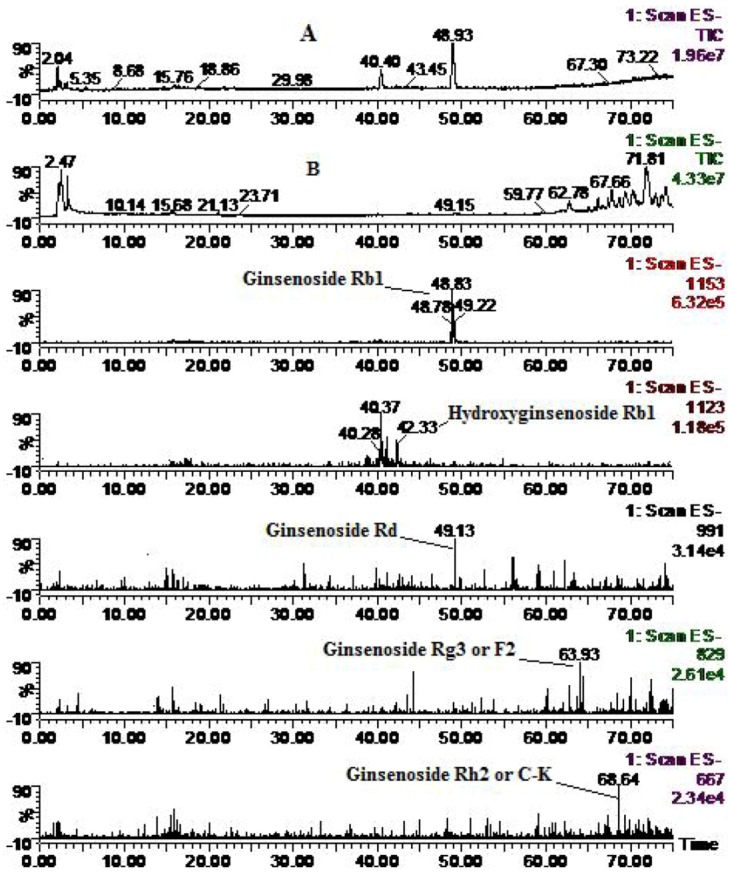
Total ion chromatogram (**A**: solution; and **B**: zebrafish) and extracted ion chromatograms for ginsenoside Rb_1_ after zebrafish exposure for 24 h.

Four metabolites of Rb_1_ were identified in addition to the parent component ginsenoside Rb_1_ (MW 1108) (Figure 4), including hydroxyginsenoside Rb_1_ (MW 1124), ginsenoside Rd (MW 946) obtained from Rb_1_ *via* cleavage of one molecule of glucose, ginsenoside Rg_3_ or F_2_ (MW 784) derived from Rb_1_ *via* cleavage of two glucose moieties, and ginsenoside Rh_2_ or C-K (MW 622), a monoglucosylated protopanaxadiol ginsenoside, derived from Rb_1_ *via* cleavage of three glucose moieties. These results were consistent with previous reports on rat metabolism [25,28].

**Figure 5:**
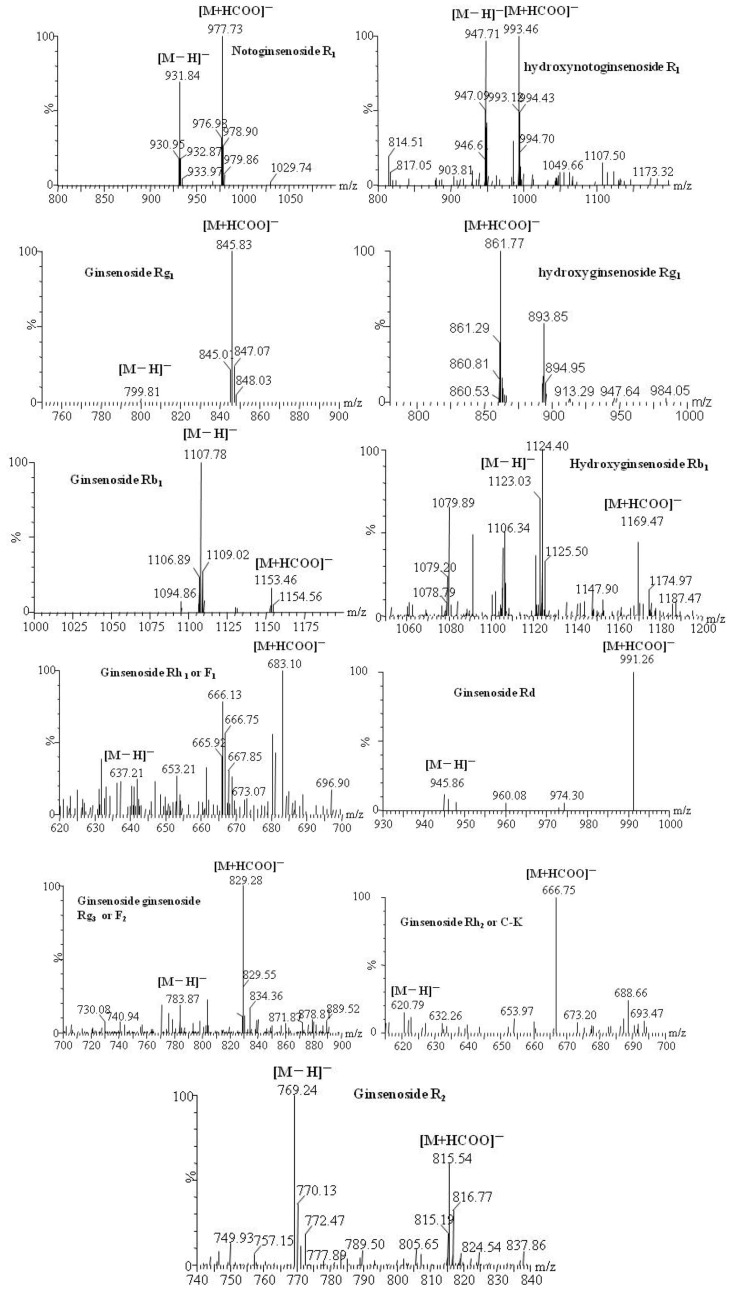
Representative MS spectra of notoginsenoside R_1_, ginsenoside Rg_1_ and ginsenoside Rb_1_ and their transformative components by zebrafish.

**Figure 6:**
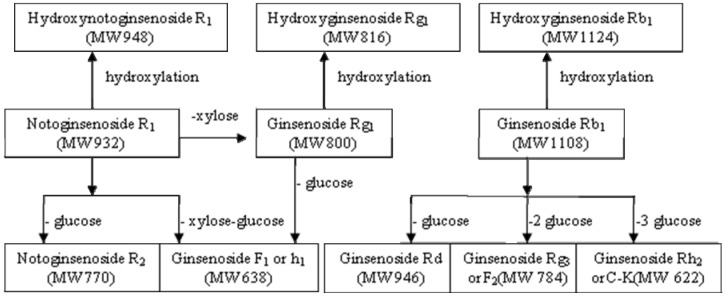
The possible metabolic pathways of notoginsenoside R_1_, ginsenoside Rg_1_ and ginsenoside Rb_1_ by zebrafish.

MS data for R_1_, Rg_1_, Rb_1_ and their metabolites by zebrafish are shown in Table 1, the representative MS spectra are shown in Figure 5, and the possible metabolic pathways of R_1_, Rg_1_ and Rb_1_ are elucidated in Figure 6.

**Table 1:** MS data for R_1_, Rg_1_ and Rb_1_ and their metabolites after zebrafish exposure for 24 h.

**Compounds**	**Retention time (min)**	**quasi-molecular ions peak**		**MW**	**Metabolite presumed**	**Zebrafish **		**Mammalian metabolism (references)**
		[M−H]^−^	[M+HCOO]^−^			**solution**	**body**	
R_1_	21.91	931.84	977.73	932.8	Notoginsenoside R_1_	+	+	[14,15,16]
	13.91	947.71	993.46	948.7	Hydroxynotoginsenoside R_1_	+		[14]
	25.98	799.81	845.83	800.8	Ginsenoside Rg_1_	+		[14, 16]
	48.07	769.24	815.54	770.2	Notoginsenoside R_2_	+		[15]
	68.52	637.21	683.1	638.2	Ginsenoside F_1_ or Rh_1_		+	[14,15,16]
Rg_1_	26.1	799.81	845.83	800.8	Ginsenoside Rg_1_	+	+	[15,16,17,18,19]
	13.06		861.77	816.8	Hydroxyginsenoside Rg_1_	+		[29]
	55.31	637.69	683.23	638.7	Ginsenoside F_1_ or Rh_1_	+		[17,18,19,20,21]
Rb_1_	48.93	1107.78	1153.46	1108.9	Ginsenoside Rb_1_	+	+	[25, 28]
	42.16	1123.03	1169.47	1124	Hydroxyginsenoside Rb_1_	+		[28]
	49.13	944.86	991.26	946	Ginsenoside Rd	+	+	[25, 28]
	63.98	783.87	829.98	784.8	Ginsenoside Rg_3_ or F_2_	+		[25, 28]
	68.66	620.79	666.75	622	Ginsenoside Rh_2_ or C-K	+	+	[25, 28]

+ detected.

### 2.3. Rationality and Advantages of Metabolic Study with Zebrafish Compared to Existing Model

The metabolic mechanisms of R_1_, Rg_1_ and Rb_1_ by zebrafish are highly consistent with the existing mammal results; over the past thirty years, a number of *in vivo* or *in vitro* metabolic studies of R_1_, Rg_1_ and Rb_1_ have been reported, though results of different reports may not consistent with each other totally, the metabolites are similar or complementary, and their metabolic mechanisms can be elucidated as deglycosylation and hydroxylation, among which deglycosylation is the major metabolic pathway of intestinal bacteria, and secondary glycoside metabolites *via* cleavage of one or multiple sugar moieties were reported; hydroxylation is another metabolic pathway, such as seen in the metabolites of hydroxynotoginsenoside R_1_ [15] and hydroxyginsenoside Rb_1_ [28]. A metabolic study of Fufang Danshen prescription presumed hydroxylation of ginsenoside Rg_1_ [29]. Our present study found deglycosylation and hydroxylation of R_1_, Rg_1_ and Rb_1_ by zebrafish for the first time, which is highly consistent with the results of existing methods: all stepwise deglycosylation metabolites were found, except protopanaxatriol (ppt) and protopanaxadiol (ppd), the reasons maybe that stable monoglucosylated ginsenoside is difficult to degrade further [30]; hydroxylation of R_1_ and Rb_1_ were totally consistent with the rat metabolism data [15,28], and hydroxylation of Rg_1_ found for the first time provides supporting evidence for the presumption of reference [29]. The results of the present study indicated that metabolism of R_1_, Rg_1_ and Rb_1_ with zebrafish is practical and feasible.

Metabolism studies with zebrafish have significant advantages of lower cost, far less amount of compound needed, easier set up and higher performance: zebrafish are small, inexpensive to maintain and easily bred in large numbers, and maintenance costs are considerably lower than those for mammals; metabolism experiments using zebrafish can be performed in ordinary laboratories under simple conditions instead of specific animal housing, metabolism cages, and high standard conditions of *in vitro* experiments, *etc.*; compared with sampling blood, bile, fence and urine of mammalian metabolism, sampling solution and fishbody of zebrafish metabolism is much more simple and easier to master, which make zebrafish experiments more efficient with much lower labor intensity. In addition, only small amounts (mg) of compounds are required, about one percent usage of rat metabolism, which make it possible for *in vivo* metabolism study of large number of trace components.

## 3. Experimental

### 3.1. Chemicals and Reagents

Notoginsenoside R_1_, ginsenoside Rg_1_, ginsenoside Rb_1_ were purchased from the National Institute for the Control of Pharmaceutical and Biological Products (Beijing, China), and their purities were all >98%. HPLC grade acetonitrile was purchased from Tedia Company (Fairfield, CT, USA), deionized water was purified by Milli-Q system (Millipore, Bedford, MA, USA), robust purified water, physiological saline (sodium chloride injection) were from Nanjing Xiaoying Pharmaceutical Group Co. Ltd. (Nanjing, China), dimethyl sulfoxide (DMSO) from Sinopharm Chemical Reagent Co. Ltd (Shanghai, China), and the other reagents were of analytical grade.

### 3.2. Animals

The adult zebrafish (*D. rerio*) of mixed sex were provided by Model Animal Research Center of Nanjing University (Nanjing, China), and acclimatized to tap water in a glass aquarium for at least 10 days prior to experimentation. Fish were kept at a temperature of 25 ± 1 °C in a photoperiod of 12:12 h. The fish were fed daily during the acclimatization period, and were fasted overnight before the day of the experiment.

### 3.3. Instruments

A Waters Alliance 2695-ZQ 2000 HPLC-MS system 2695 liquid chromatography system (Waters Corporation, milford, MA, USA) consisting of a quadruple pump, an autosampler, column temperature controller and a PDA detector, Micromass ZQ2000 single-quadrupole mass spectrometer (Waters) with an electrospray ionization source, Masslynx 4.0 ChemStation software; Mettler Toledo AB135-S Analytical Balance (Mettler Toledo, schwerzenbach, Switzerland); KQ3200DE Digital Ultrasonic Washer (Kunshan Ultrasonic Instruments Co. Ltd, Kunshan, China); Labconco Freezer Dryer (Labconco, kansas, MO, USA); TGL-16G Desk Centrifuge (Shanghai Anting Scientific Instrument Factory, Shanghai, China), Organomation N-EVAP^TM^ 112 Nitrogen Evaporator (Organomation Associates, Inc. berlin, MA, USA).

### 3.4. Biological Sample Collection

Adult zebrafish were divided into four experimental groups of five fish each after fasting for 12 h one blank control group was exposed to 1% DMSO purified water (blank zebrafish group), three groups were exposed to 30 mL solution of R_1_ (9.63 µg/mL), Rg_1_ (16.75 µg/mL) and Rb_1_ (21.24 µg/mL) in 1% DMSO purified water (drug zebrafish groups), respectively. In addition, the above solutions of R_1_ (9.63 µg/ml), Rg_1_ (16.75 µg/mL) and Rb_1_ (21.24 µg/mL) without zebrafish were used as blank drug controls. Zebrafish of the blank zebrafish group and drug zebrafish groups were sampled at 24 h, respectively, and the zebrafish body samples of each group were combined and washed rapidly with 1% DMSO purified water three times and stored at −70 °C prior to analysis; Solution of blank zebrafish group and drug zebrafish groups at 24 h were combined, respectively, 8 mL (n = 3) of solution of each group were sampled and also stored at −70 °C prior to analysis. Blank drug control solution (8 mL, n = 3) were sampled as above at 0 h, 24 h.

### 3.5. Sample Preparation

The solution sample (8 mL) was freeze-dried to dryness, and the residue was dissolved in 1 mL 90% methanol. After centrifugation at 15,000 rpm for 15 min, 20 μL of the supernatant was introduced into the HPLC-MS system for analysis. The zebrafish body sample (five fish of each group) were cut with scissorc, and 1 g was sampled and homogenized with physiological saline (5 mL), followed by centrifugation at 3,500 rpm for 15 min, the supernatant was suspended with methanol (20 mL), and vortex mixed, followed by centrifugation at 3,500 rpm for 15 min. The supernatant was evaporated to dryness with nitrogen at 40 °C, and the residue was dissolved in 90% methanol with the final content of 1 g zebrafish/mL. After centrifugation at 15,000 rpm for 10 min, 20 μL of the supernatant was injected into the HP LC-MS system for analysis.

### 3.6. Analysis Condition

HPLC–MS was performed with a Waters Alliance 2695 - ZQ 2000 single - quadrupole mass spectrometer equipped with an electrospray ionization source. The HPLC analysis was carried out on the column configuration consisted of an Agilent Zorbax Extend reversed - phase C_18_ column (5 mm, 250 mm × 4.6 mm) and an Agilent Zorbax extend - C_18_ guard column (5 mm, 20 mm × 4 mm). The column was maintained at 30 °C, the flow rate was 1.0 mL/min. A gradient elution of 0.05% aqueous formic acid (A) and 0.05% acetonitrile formic acid (B) was used as 7–17% B at 0–10 min, 17–20% B at 10–12 min, 20–21% B at 12–16 min, 21% B at 16–32 min, 21–29% B at 32–40 min, 29–35% B at 40–55 min, 35–65% B at 55–65 min, 65–80% B at 65–80 min, and 80% B at 70–75 min. The mass spectra were recorded with full scan mode in negative mode, capillary voltage 2.5 kV, cone voltage 35 V, drying gas flow rate 320 L/h, ion source temperature 120 °C, adjuvant gas temperature 310 °C, mass range 100~1200 *m/z*, extract ion current (TIC): [M−H]^−^; [M+HCOO]^−^.

## 4. Conclusions

We have demonstrated for the first time the feasibility of the metabolic study of microamounts of notoginsenoside R_1_ (R_1_), ginsenoside Rg_1_ (Rg_1_) and ginsenoside Rb_1_ (Rb_1_), which are components isolated from Panax notoginseng, using a zebrafish model, and metabolic information could be nicely identified with HPLC-ESI-MS. The results showed that metabolic products of R_1_, Rg_1_ and Rb_1_ resulting from deglycosylation and hydroxylation in zebrafish were highly consistent with those from metabolism of mammals, which confirmed our hypothesis that metabolism studies of compounds from TCMs with the proposed zebrafish model is possible and reasonable. With the advantages of lower cost, easier set up and higher performance, the zebrafish metabolic model may become a novel, powerful model for quick predication on drug metabolism, especially for those trace compounds which could greatly enrich our current knowledge of the metabolism models of TCMs.
